# Gilteritinib Monotherapy as a Transplant Bridging Option for a Patient with *FLT3*-Mutated Acute Promyelocytic Leukemia Who Developed a Second Relapse after All-Trans Retinoic Acid + Chemotherapy, Arsenic Trioxide, and High-Dose Cytarabine Therapy

**DOI:** 10.1155/2023/8568587

**Published:** 2023-12-13

**Authors:** Hirofumi Kobayashi, Hiroki Tsutsumi, Yukiko Misaki, Takashi Maekawa, Naoko Inoshita, Machiko Kawamura, Nobuo Maseki

**Affiliations:** ^1^Department of Hematology, Saitama Cancer Center, Saitama, Japan; ^2^Division of Hematology, Jichi Medical University Saitama Medical Center, Saitama, Japan; ^3^Department of Pathology, Saitama Cancer Center, Saitama, Japan; ^4^Department of Pathology, Moriyama Memorial Hospital, Tokyo, Japan; ^5^Department of Clinical Laboratory Medicine, Saitama Cancer Center, Saitama, Japan

## Abstract

We report a case of FLT3-mutated APL who developed disease relapse despite all-trans retinoic acid (ATRA) + chemotherapy, and re-induction chemotherapy with arsenic trioxide (ATO) and high-dose (HD) cytarabine (Ara-C) therapy failed to yield complete remission. Because the leukemic cells were resistant to all the aforementioned therapies, we started the patient on monotherapy with gilteritinib, a selective FLT3-inhibitor, as an alternative re-induction treatment option rather than further intensive chemotherapy. The patient showed complete hematologic remission in response to this therapy. This case serves as supporting evidence for the use of single-agent therapy with gilteritinib as a bridge to transplantation in patients with refractory *FLT3*-mutated APL.

## 1. Introduction

Acute promyelocytic leukemia (APL) is characterized by the presence of the *t* (15; 17) chromosomal abnormality and the promyelocytic leukemia (PML), retinoic acid receptor alpha (RAR*α*) fusion protein within the cells [1]. All-trans retinoic acid (ATRA) + chemotherapy has been used as the standard treatment regimen for APL [1]. The combination of arsenic trioxide (ATO) and ATRA is not yet approved in Japan.

The presence of internal tandem duplication (ITD) in the juxta membrane region of the fms-related receptor tyrosine kinase 3 (*FLT3*) gene is the most common genomic alteration in acute myeloid leukemia (AML) and is known to be associated with a poor prognosis [2]. Allogeneic stem cell transplantation (alloHSCT) performed after the first complete remission (CR) substantially improves the otherwise dismal outcomes [3]. Use of an FLT3 inhibitor as bridging therapy to transplantation if necessary and as maintenance therapy before/after alloHSCT has become the standard of care in AML patients with an *FLT3* mutation detected at diagnosis [3].

Approximately 20%–27% of cases of APL are reported to carry *FLT3*-ITDs, which are known to be associated with high peripheral white blood cell (WBC) counts and higher early death (ED) rates [4, 5]. Use of FLT3 inhibitors for the treatment of APL, however, has not been discussed so far.

We describe the case of a patient with APL harboring *t* (15; 17) and an *FLT3*-ITD who received treatment at our institution between March 2020 and April 2022. She was treated for relapsed and persistent disease and bridged successfully to alloHSCT using gilteritinib monotherapy.

## 2. Case Presentation

A previously healthy 57-year-old woman presented to her local hospital with severe genital bleeding, anemia, and thrombocytopenia (hemoglobin 7.0 g/L and platelet (PLT) count 53 × 10^9^/L). The total WBC count was 74.2 × 10^9^/L, with the blast cell count of 67.5 × 10^9^/L. She was transferred to our hospital on the same day. Bone marrow aspiration revealed 87.8% myeloid blasts, with marked nuclear malformations and Faggot cells. G-banding revealed *t* (15; 17) (q22; q21) in all the cells were examined. A *PML::RARA* fusion messenger RNA was detected by real-time polymerase chain reaction (PCR), and an *FLT3*-ITD was also detected. The leukemic cells showed positive immunohistochemical staining for CD2, CD13, CD33, and CD34 and negative staining for HLA-DR and CD56.

Treatment using the standard regimen of ATRA, idarubicin, and cytarabine (Ara-C) yielded complete remission of the disease. Consolidation therapies with daunorubicin, mitoxantrone, idarubicin, and Ara-C were administered sequentially, as mentioned [6]. Although no neurological abnormalities were observed, cerebrospinal fluid examination revealed a cell count of 11/*μ*L and the cytology was categorized as class V, which led us to make a diagnosis of central nervous system (CNS) leukemia. After two intrathecal administrations of 15 mg methotrexate (MTX) and 30 mg Ara-C, the cerebrospinal fluid cell count improved to less than 1/*μ*L and the cytology to class II. The drugs were administered intrathecally at the same doses a total of six times, and the CNS leukemia resolved. Minimal residual disease (MRD) in the peripheral blood also became negative after 3 months of treatment.

However, despite receiving maintenance therapy with ATRA, the patient developed disease relapse 14 months after the initial diagnosis. The variant allele frequency (VAF) of the *FLT3*-ITD in the bone marrow cells was 79%. We initiated chemotherapy with ATO, but this treatment was discontinued twice due to prolongation of the corrected QT (QTc) interval and its failure to yield remission.

Therefore, we started the patient on chemotherapy with tamibarotene. Bone marrow aspiration revealed complete remission of the disease after 2 months of chemotherapy. Because MRD in the bone marrow failed to resolve, autologous HSCT was not selected as consolidation therapy, and we registered the patient in the Japan marrow donor program (JMDP).

Then, we started the patient on high-dose (HD) Ara-C therapy 56 days prior to the start of gilteritinib administration, which was, however, followed by a second relapse of her disease. The WBC count was elevated to 4.22 × 10^9^/L with 7% blasts, and the PLT count had decreased to 39 × 10^9^/L. Because no recommended therapeutic option remained, except for gemtuzumab ozogamicin (GO), which has been known to increase the risk of veno/sinusoidal obstructive disease (VOD/SOS) during HSCT, we decided to try gilteritinib monotherapy.

**Figure 1:**
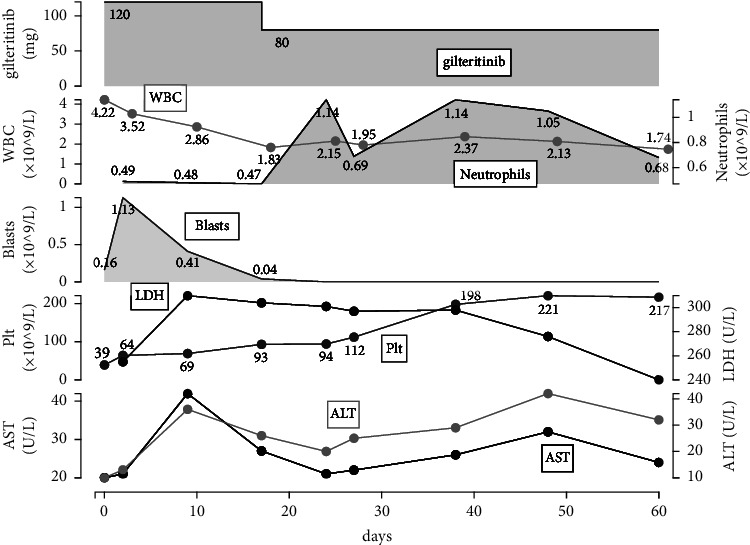
Clinical course of the patient during administration of gilteritinib. The details are described in the case report. WBC: white blood cell; Plt: platelet; AST: aspartate aminotransferase; ALT: alanine aminotransferase; LDH: lactate dehydrogenase.

Remission of the bone marrow disease was obtained after 60 days of treatment. The clinical course of the patient during the treatment with gilteritinib is shown in Figure 1. We started the patient on gilteritinib at the dose of 120 mg, which was prophylactically reduced after 17 days of treatment to 80 mg, to avoid the necessity for discontinuation due to QTc prolongation. The QTc gradually decreased and remained at less than 450 milliseconds (ms) from day 11 after the dose reduction through the entire course of gilteritinib administration. Peripheral blood blasts disappeared after 18 days. After 9 days, no platelet transfusion was required, and the platelet count became normal after 39 days. Although the bone marrow was in remission, MRD was positive in the bone marrow cells.

Allogeneic peripheral blood stem cells were infused after pretreatment with fludarabine, busulfan, and thymoglobulin plus total body irradiation at 4 Gy, which were started 3 days after the end of gilteritinib administration. The stem cells became engrafted on day 19, and blood transfusion became unnecessary after day 15. A rash appeared and expanded over the body from day 19 after alloHSCT. Biopsy revealed the diagnosis of graft-versus-host disease (GvHD). The skin GvHD worsened up to stage 3, grade II. Because the GvHD was limited to the skin, we did not administer systemic steroid therapy. From day 25, the patient began to experience headache, nausea, and anorexia. Cerebrospinal fluid examination and nuclear magnetic resonance imaging (MRI) revealed no abnormalities. She developed fever on day 43 and severe back pain from the shoulder region that necessitated administration of opioids. Delirium appeared; however, MRI still revealed no abnormalities. Recurrence of leukemia was suspected, but blood and cerebrospinal fluid findings failed to lend support to the suspicion of recurrence. Ebstein–Barr virus (EBV) DNA was detected at 4.2 × 10^5^ copies/mL in a blood specimen collected on day 46. Hepatic and renal function impairment also continued to progress, with diuretics proving ineffective, finally resulting in anuria. The patient died of multiple organ failure on day 49.

At autopsy, EBV-positive B cells were found to have invaded various organs throughout the body, including the lungs, liver, and kidneys, and diagnosis of post-transplant lymphoproliferative disorder was made. Post-treatment, the skin GvHD was still active. There were no findings at autopsy suggesting the persistence of leukemic cells. The cause of death was thought to be systemic organ failure due to post-transplant lymphoproliferative disorder.

## 3. Discussion

Our experience in this case suggests that gilteritinib monotherapy can be used to achieve complete remission in APL patients harboring *t* (15; 17) and *FLT3* mutations; the treatment was well-tolerated and associated with significantly lower toxicity than the current standard-of-care consolidation high-dose chemotherapy regimens. This is the first case report of administration of gilteritinib as a bridging therapy to transplantation in a patient with *FLT3*-mutated relapsed/refractory APL. Although molecular remission was not obtained in our case, alloHSCT was successfully performed and no leukemic cells were detected at autopsy on day 49. Another therapeutic option may be venetoclax, a selective inhibitor of B-cell lymphoma 2 (BCL-2), in combination with a hypomethylating agent (HMA), azacytidine, but this regimen is also of limited use for patients with APL [7].

Song et al. reported that the complete remission (CR) and differentiation syndrome (DS) rates in APL patients with and without *FLT3*-ITDs were similar, while Kaplan–Meier analysis of the 5-year event-free survival (EFS) and overall survival (OS) rates were significantly different between the patient groups stratified by the *FLT3*-ITD mutation status [4]. They speculated that the ATRA + ATO dual-induction regimen could eliminate the adverse prognosis associated with *FLT3*-ITDs. Because the ATRA + ATO dual-induction regimen is still not approved in Japan, the induction therapy was based on ARTA + chemotherapy and ATO was administered after relapse, but the treatments proved ineffective.

This patient reported here showed CNS infiltration at diagnosis. Cerebrospinal fluid leukemia is not common in patients with APL without CD56-positivity [8], whereas it is frequently encountered in patients with AML harboring *FLT3*-ITDs [9].

The ADMIRAL trial reported a significantly longer median overall survival in patients who had received gilteritinib after transplantation that in patients who had not received gilteritinib after transplantation [10]. Those who showed a response and underwent subsequent transplantation were continued in the trial, and gilteritinib therapy was resumed 30 to 90 days after the transplantation if engraftment had occurred in the absence of disease relapse and there were no uncontrolled complications of transplantation [11]. Although stem cell engraftment was achieved in our patient, we were unable to restart gilteritinib because her general condition had deteriorated before day 30 after the transplantation.

Our patient showed hematologic remission in response to gilteritinib monotherapy, without any serious complications, and this treatment allowed the patient to be bridged successfully to alloHSCT, although she had persistent MRD. Studies to investigate the promise of gilteritinib in combination with HMA therapy or with conventional induction chemotherapy and as maintenance therapy following achievement of the first remission are ongoing [2]. Although chemotherapy with venetoclax in combination with azacytidine could be another treatment option for APL patients showing relapse after ATRA and ATO therapy, the validity of using gilteritinib alone or in combination with other agents needs to be confirmed.

## Data Availability

The data used to support the findings of this case report are included within the article.
